# Effect of Different Light Spectrum in *Helicoverpa armigera* Larvae during HearNPV Induced Tree-Top Disease

**DOI:** 10.3390/insects9040183

**Published:** 2018-12-04

**Authors:** Mandira Katuwal Bhattarai, Upendra Raj Bhattarai, Ji-nian Feng, Dun Wang

**Affiliations:** 1 State Key Laboratory of Crop Stress Biology for Arid Areas, Northwest A&F University, Yangling 712100, China; mandirakat123@gmail.com (M.K.B.); upendrarajbhattarai@gmail.com (U.R.B.); 2 Department of Entomology, Northwest A&F University, Yangling 712100, China

**Keywords:** baculovirus, behavior manipulation, *Helicoverpa armigera* larvae, phototaxis, tree-top disease

## Abstract

Lepidopteran larvae upon infection by baculovirus show positive photo-tactic movement during tree-top disease. In light of many insects exploiting specific spectral information for the different behavioral decision, each spectral wavelength of light is an individual parsimonious candidate for such behavior stimulation. Here, we investigated the responses of third instar *Helicoverpa armigera* larvae infected by *Helicoverpa armigera* nucleopolyhedrovirus (HearNPV) to white (broad-spectrum), blue (450–490 nm), UVA (320–400 nm), and UVB (290–320 nm) lights for the tree-top disease. Our findings suggest that tree-top phenomenon is induced only when the light is applied from above. Blue, white and UVA lights from above induced tree-top disease, causing infected larvae to die in an elevated position compared to those larvae living in the complete dark. In contrast, UVB from above did not induce tree-top disease. Blue light exerted the maximum photo-tactic response, significantly (*p* < 0.01) higher than white light. The magnitude of the response decreased with decreasing wavelength to UVA, and no response at UVB. Our results suggested that the spectral wavelength of the light has a significant effect on the induction of the tree-top disease in *H. armigera* third instar larvae infected with HearNPV.

## 1. Introduction

Behavior manipulation in the host-parasite interaction has been defined as the phenotypic changes in the host organism [1]. It has evolved as one of the primary tools in all major phylogenetic lineages of parasites and been efficiently utilized to perpetuate their genes [2,3,4,5]. Environmental factors hold a significant role together with molecular cross-talks between species for modulating the phenotypes of an organism [6]. Sensory stimuli like temperature, light, and gravity have been found to play important roles in regulating behaviors like locomotion, mate choice, reproduction etc. [7,8,9,10]. Being such a common phenomenon, the associated mechanisms are just beginning to be deciphered.

Baculoviruses are host specific pathogens associated with arthropods and mainly infect lepidopteran larvae [11]. Induced behaviors in lepidopteron host larvae by their pathogenic baculoviruses have been a field of extensive research since few decades. Two kinds of altered behaviors in the infected larvae have been reported and are governed by different triggering mechanisms in the baculovirus [12]. Firstly, the enhanced locomotory activity (ELA), where larvae become hyperactive [13,14] and secondly, the tree-top disease (*Wipfelkrankheit*) where, infected larvae climb high up to the top of the tree before death, resulting in the better dispersal of virus particles with broader foliage cover after liquefaction, thus increasing chances of virus transmission to subsequent host larvae [15,16].

Hoover, Grove [16] reported that the ecdysteroid UDP-glucosyl transferase (*egt*) gene of *Lymantria dispar* multi-nucleopolyhedrovirus (LdMNPV) triggers the tree-top disease in *L. dispar* larvae. In addition, *Autographa californica* MNPV was found to induce similar disease in both *Trichoplusia ni* and *Spodoptera exigua* larvae but the *S. exigua* larvae showed a molting dependent effect. Ros, van Houte [17] reported that those *S. exigua* larvae undergoing molting during infection showed the tree-top disease, and vice versa. They further reported that, the mutant AcMNPV lacking the *egt* gene did not affect the position of larval death. Similarly, in SeMNPV–*S. exigua* interaction, *egt* was involved indirectly in tree-top disease by prolonging the lifespan of infected larvae [18]. These findings suggest that the effect of *egt* on the tree-top disease is not conserved among all baculoviruses.

The role of light as a key factor in the induction of tree-top disease is a recently discovered avenue. The *S. exigua* larvae infected with SeMNPV were positively photo-tactic [19] with the need for the light in a particular time frame for triggering the tree-top disease [20]. The varying responses of different insect species upon exposure to different light wavelengths on their physiological, biological and behavioral aspects have previously been reported [21,22,23,24]. For example, the violet light attracts *Orius sauteri* (Poppius) but not *Trialeurodes vaporariorum* [23,25]. *Tetranychus urticae* avoids UVA (320–340 nm) but *Aphis fabae* is attracted to ultraviolet (UV) light (<400 nm) [26,27]. The UVC possess sub-lethal to lethal effects on *Bombyx mori*, *Callosobruchus maculatus*, *Dermatophagoides pteronyssinus*, *D. farina* [28,29,30]. Similarly, the UVB shows a lethal effect on *T. urticae* eggs but no any effect from UVA is reported [27]. The blue light (467 nm) causes mortality in the pupae of *T. confusum* and *Drosophila melanogaster* [31]. Therefore, we believe it is integral to clarify the importance and involvement of different wavelengths of light in an apparent photo-tactic effect during the tree-top disease in lepidopteran larvae.

For the abovementioned reason, we investigated the effect of different wavelengths of lights categorically: White (broad-spectrum), Blue (450–490 nm), UVA (320–400 nm), and UVB (290–320 nm) in the third instar larvae of *Helicoverpa armigera* Hübner (Lepidoptera: Noctuidae) infected with HearNPV from pre-symptomatic to tree-top disease stage.

## 2. Materials and Methods

### 2.1. Insect Larvae and Virus

Larvae of *Helicoverpa armigera* (Hübner) were reared on a wheat bran rich artificial diet at temperature 28 ± 1 °C, 50% relative humidity and the photoperiod of 12 L: 12 D (Light: Dark) [32]. HearNPV viral occlusion bodies (OB) were amplified in *H. armigera* 4th instar larvae; collected, purified, quantified and stored at 4 °C as described previously [33].

### 2.2. Behavioral Assay 1: Light from Above

Newly molted third instar larvae were starved for 24 h and fed with the viral dose of LD90 (2800 OBs/larvae) [33]. Infection was done following a modified droplet feeding method [14]. Briefly, the prepared virus stock (2800 OBs/µL) was mixed with equal volume of 10% sucrose solution with green colored food dye (Shaanxi Top Pharm Chemical Co., Ltd., Xi’an, China) and 2 µL of the prepared inoculant was placed at the bottom of the 1.5 µL centrifuge tubes. Each larva was guided inside those tubes by placing them upside down. By 5 min most larvae had finished ingesting the provided virus suspension by climbing up the tube. The larvae which finished ingesting the liquid were then transferred individually in a plastic column of 6 cm diameter and 28 cm height, lined with mesh wire in the inner surface and transparent plastic wrap with 6 small holes equipped for ventilation at both ends. The diet cube (approx. 3 cm^3^) was placed at the bottom of the cylinder. The sides and bottom of the cylinders were wrapped with aluminum foil and placed in the black box in order to block light from other directions. Treatment consisted of 4 different lights: White light (broad-spectrum), Blue light (450–490 nm), UVA (320–400 nm), and UVB (290–320 nm) placed 30 cm above the cylinders for luminescence during light hours of the day (12 L: 12 D). Similarly, treated larvae were kept in a complete dark (0 L: 24 D) as a control treatment. Mock-treated larvae were also subjected to similar treatments. Each experiment was conducted with three independent biological replicates and each replicate consisted of 15 larvae.

The vertical position of mock-infected larvae was recorded manually using a measuring scale twice a day till pupation; a red light was used to measure the vertical position of mock-infected larvae in the dark condition. The top vertical position where the infected larva was found dead was recorded at 8-days post-infection (dpi). Death from baculovirus infection was further confirmed by screening for liquefaction of the larvae following death, and those who did not die or survived despite being fed with the virus were excluded from the data analyses.

### 2.3. Behavioral Assay 2: Light from Below

To investigate the effect of direction of light in assay 1, a similar experiment was repeated following the same experimental setup except the aluminum foil on the cylinder was wrapped on the side and the top, excluding the bottom, and all the lights used in the treatments were applied from the bottom at a 30 cm distance from the cylinder placed inside the black box. In addition, control treatment consisted of a similar setup with mock-infected larvae as above. Data were recorded in a similar manner as in assay 1.

### 2.4. Statistical Analysis

Data obtained regarding the height of the larvae at death were analyzed using the linear regression model in R (v3.0.0). The regression model was fitted using treatments (different lights) and replications as explanatory variables and the height of death as a dependent variable. Most of the larvae died during its fourth instar so were excluded as an explanatory variable [20].

## 3. Results

### 3.1. Different Lights from Above Affecting Tree-Top Disease

**Figure 1:**
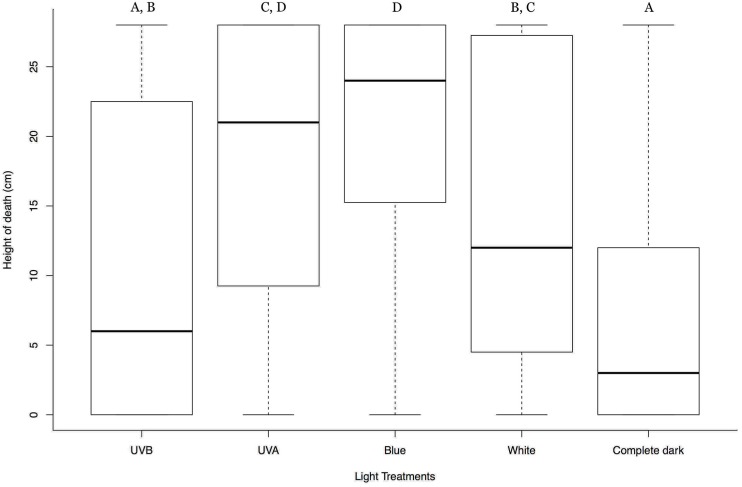
Box plots summarizing the vertical position of the HearNPV infected *H. armigera* larvae at death in different light treatments from above. Y-axis represents the vertical position of the dead larvae from the base of the cylinder in centimeter (cm); X-axis represents different light treatments; In each box plot, the heavy horizontal line crossing the box is the median, the bottom, and top of the box are the lower and upper quartiles, and the whiskers are the minimum and maximum values. Clusters with the same letter code are not significantly different.

Results showed that the larvae with white, blue, and UVA light treatment died at a significantly higher position than that from the complete dark treatment; *t*-test = 4.163, 7.627, 6.119 respectively; d.f. = 369; *p* < 0.0001. There was also a significant difference between the height of death of the larvae between the blue light and the white light treatment (*t*-test = −3.493; d.f. = 369; *p* = 0.00484); blue light causing the larvae to die at a more elevated position than the white light. Treatment with UVB light however, did not significantly affect the larval position at death compared to that of the complete dark; *t*-test = 1.728; d.f. = 369; *p* = 0.0849. No significant differences were found between the replicates (*t*-test = 0.201; d.f. = 369; *p* = 0.841) (Figure 1). Mock-infected larvae did not illustrate any differences regarding climbing behavior between treatments. They showed various climbing peaks irrespective of light conditions and then gradually descended to the bottom close to pupation (Supplementary Figures S1–S5).

### 3.2. Effect of Lights from Below on Tree-Top Disease

**Figure 2:**
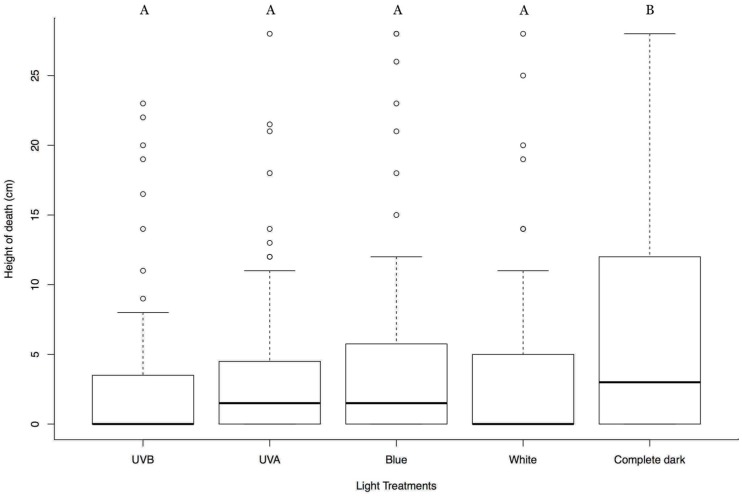
Box plots summarizing the vertical position of the HearNPV infected *H. armigera* larvae at death in different light treatments from below. Y-axis represents the vertical position of the dead larvae from the base of the cylinder in centimeters (cm); X-axis represents different light treatments; In each box plot, the heavy horizontal line crossing the box is the median, the bottom, and top of the box are the lower and upper quartiles, the whiskers are the minimum and maximum values, and the dots are the outliers. Clusters with the same letter code are not significantly different.

To see the effect of direction of light on the tree-top disease we applied all the different spectrum of lights in a similar way but from below. Result showed that the white, blue, UVA, and UVB lights from below caused infected larvae to die at significant lower height compared to that of complete dark (*t*-test = −3.428, −2.852, −3.378, −3.168 respectively; d.f. = 349; *p* < 0.001, <0.01, <0.001, <0.001 respectively) (Figure 2). This signifies that, the infected larvae react to the direction of the light and light coming from above can only induce tree-top disease. No any statistical differences were observed between the replicates (*t*-test = 1.652; d.f. = 349; *p* = 0.1). Mock-infected larvae did not show any difference in climbing behavior between treatments, instead, they showed similar patterns of multiple peaks, and all of them descended to the bottom near pupation (Supplementary Figures S5–S9).

## 4. Discussion

The ecological and evolutionary significance of baculovirus induced behavior changes in lepidopteran larva have led to some exciting discoveries adding to the fundamental understanding of the mechanisms involved. Various studies have revealed the involvement of baculovirus gene (*egt*), and the need for light from above during a certain time period of infection for the induction of the tree-top disease [16,17,18,20]. In this study, we investigated the importance of lights of different wavelength categories: White light (broad-spectrum), blue light (450–490 nm), UVA (320–400 nm), and UVB (290–320 nm) in relation to climbing before death behavior in HearNPV infected *H. armigera* third instar larvae, and surprisingly found that white light along with blue and UVA applied from above induced tree-top disease, but UVB did not. This striking difference on the wavelength sensitivity and their resultant effect on the photomovement of infected larvae during tree-top disease holds a significant ecological importance because of the fact that viruses are the one that lay behind the strings for this photomovement [12,16] and exposure to ultraviolet light especially UVB spectrum can severely limit the virulence and pathogenicity of the baculovirus [34,35,36]. Although, baculoviruses inside the insect cells can garner some protection from UVB due to the physical barrier or a repair mechanism of the host cells but still are found to be much sensitive upon longer exposure [37] which signifies the importance of wavelength dependent phototaxis effect during the tree-top disease.

Our observations showed that light from above is essential for tree-top disease and this is in accordance with previous findings [20,38]. Our efforts to further dissect the effect and preference of different wavelengths of light during the process unveiled the higher preference of infected larvae toward the blue light coming from above. And the degree of effect of light decreases as the wavelength decreases to UVA and finally stops with further shortening the wavelength to UVB (Figure 1). In addition, the wide spectrum white light showed a comparatively lesser effect on the photomovement over the blue light. The differential behavioral response of the host harboring pathogens to various components of lights has also been noticed in other host pathogen-interaction [39].

Phototaxis mechanism has been observed in many insect species [40,41,42,43]. Their preference for specific wavelengths differs between species and also between developmental stages [44,45,46]. Wavelength preference, particularly for blue spectra has been described in several species like *Drosophila*, *Euglena gracilis*, *Cyanobacterium*, Frog [47,48,49,50,51]. Similarly, adult lepidopteran insects were also found to be attracted toward blue or shorter wavelengths of light, and that might assist them with orientation cues especially for finding suitable oviposition sites which is not the case for their larvae [44,52]. Therefore, the infected larva’s high preference for the blue light might have indicated their search for a place before death, rather than the search for leaves in the canopy for food because the infected ones consume less food and have reduced growth rate and development [38].

## 5. Conclusions

These findings provide a fundamental understanding about the role of different light spectrums on climbing before death behavior of baculovirus-infected lepidopteran larvae. We found that light from above is needed to induce tree-top disease in HearNPV infected *H. armigera* larvae. Lights within the spectrum of white, blue and UVA induced the tree-top disease, whereas UVB did not, and the blue light exerted significant photo-movement compared to white light. Therefore, in-depth molecular investigation of the spectral light perception of the infected larvae during the tree-top disease can possibly outline the complete molecular mechanism of the host impetus for the before death climbing behavior.

## Supplementary Materials

The following are available online at http://www.mdpi.com/2075-4450/9/4/183/s1, Figures S1–S9. The Climbing behavior of uninfected larvae in different light treatments. Y-axis represents the vertical position of the larvae in centimeter from the base of the cylinder; X-axis represents different hour post light treatments until pupation; Error bar represents standard error mean (SEM); S1–S4 illustrates larval climbing behavior in different light treatments from below (L: D, 12 h: 12 h); S5 during complete dark (L: D, 0 h: 24 h); and S6–S9 during different light treatments from above (L: D, 12 h: 12 h) as mentioned in the title of each line graph.

## References

[1] Poulin R. (1995). Adaptive changes in the behaviour of parasitized animals: A critical review. Int. J. Parasitol..

[2] Heil M. (2016). Host Manipulation by Parasites: Cases, Patterns, and Remaining Doubts. Front. Ecol. Evol..

[3] Moore J. (2002). Parasites and the Behavior of Animals.

[4] Poulin R. (2010). Parasite Manipulation of Host Behavior: An Update and Frequently Asked Questions. Advances in the Study of Behavior.

[5] Lefèvre T., Lebarbenchon C., Gauthier-Clerc M., Missé D., Poulin R., Thomas F. (2009). The ecological significance of manipulative parasites. Trends Ecol. Evol..

[6] Lobo I., Shaw K. (2008). Phenotypic range of gene expression: Environmental influence. Nat. Educ..

[7] Cézilly F., Grégoire A., Bertin A. (2000). Conflict between co-occurring manipulative parasites? An experimental study of the joint influence of two acanthocephalan parasites on the behaviour of Gammarus pulex. Parasitology.

[8] Brodeur J., McNeil J.N. (1990). Overwintering microhabitat selection by an endoparasitoid (Hymenoptera: Aphidiidae): Induced phototactic and thigmokinetic responses in dying hosts. J. Insect Behav..

[9] Berdoy M., Webster J.P., Macdonald D.W. (2000). Fatal attraction in rats infected with *Toxoplasma gondii*. Proc. R. Soc. Lond. B Biol. Sci..

[10] Zimmer C. (2000). Parasite Rex: Inside the Bizarre World of Nature’s Most Dangerous Creatures.

[11] Williams T., Bergoin M., van Oers M.M. (2017). Diversity of large DNA viruses of invertebrates. J. Invertebr. Pathol..

[12] Van Houte S., Ros V.I.D., van Oers M.M. (2014). Hyperactivity and tree-top disease induced by the baculovirus AcMNPV in *Spodoptera exigua* larvae are governed by independent mechanisms. Naturwissenschaften.

[13] Kamita S.G., Nagasaka K., Chua J.W., Shimada T., Mita K., Kobayashi M., Maeda S., Hammock B.D. (2005). A baculovirus-encoded protein tyrosine phosphatase gene induces enhanced locomotory activity in a lepidopteran host. Proc. Natl. Acad. Sci. USA.

[14] Bhattarai U.R., Katuwal Bhattarai M., Li F., Wang D. (2018). Insights into the temporal gene expression pattern in *Lymantria dispar* larvae during the baculovirus induced hyperactive stage. Virol. Sin..

[15] Goulson D. (1997). Wipfelkrankheit: Modification of host behaviour during baculoviral infection. Oecologia.

[16] Hoover K., Grove M., Gardner M., Hughes D.P., McNeil J., Slavicek J. (2011). A gene for an extended phenotype. Science.

[17] Ros V.I.D., van Houte S., Hemerik L., van Oers M.M. (2015). Baculovirus-induced tree-top disease: How extended is the role of egt as a gene for the extended phenotype?. Mol. Ecol..

[18] Han Y., van Houte S., Drees G.F., van Oers M.M., Ros V.I.D. (2015). Parasitic Manipulation of Host Behaviour: Baculovirus SeMNPV EGT Facilitates Tree-Top Disease in *Spodoptera exigua* Larvae by Extending the Time to Death. Insects.

[19] Van Houte S., van Oers M.M., Han Y., Vlak J.M., Ros V.I.D. (2014). Baculovirus infection triggers a positive phototactic response in caterpillars to induce ‘tree-top’ disease. Biol. Lett..

[20] Han Y., van Houte S., van Oers M.M., Ros V.I.D. (2017). Timely trigger of caterpillar zombie behaviour: Temporal requirements for light in baculovirus-induced tree-top disease. Parasitology.

[21] Hariyama T., Saini R.K. (2001). Odor Bait Changes the Attractiveness of Color for the Tsetse Fly. Tropics.

[22] Shimoda M., Honda K.-I. (2013). Insect reactions to light and its applications to pest management. Appl. Entomol. Zool..

[23] Vaishampayan S.M., Kogan M., Waldbauer G.P., Woolley J.T. (1975). Spectral specific responses in the visual behavior of the greenhouse whitefly, *Trialeurodes vaporariorum* (Homoptera: Aleyrodidae). Entomol. Exp. Appl..

[24] Webb R.E., Smith F.F., Affeldt H., Thimijan R.W., Dudley R.F., Webb H.F. (1985). Trapping greenhouse whitefly with coloured surfaces: Variables affecting efficacy. Crop Prot..

[25] Ogino T., Uehara T., Muraji M., Yamaguchi T., Ichihashi T., Suzuki T., Kainoh Y., Shimoda M. (2016). Violet LED light enhances the recruitment of a thrip predator in open fields. Sci. Rep..

[26] Hardie J. (1989). Spectral specificity for targeted flight in the black bean aphid, *Aphis fabae*. J. Insect Physiol..

[27] Sakai Y., Osakabe M. (2010). Spectrum-specific Damage and Solar Ultraviolet Radiation Avoidance in the Two-spotted Spider Mite. Photochem. Photobiol..

[28] Heidari N., Sedaratian-Jahromi A., Ghane-Jahromi M. (2016). Possible effects of Ultraviolet ray (UV-C) on biological traits of *Callosobruchus maculatus* (Col.: Chrysomelidae). J. Stored Prod. Res..

[29] Nakajima M., Yoshida H. (1971). Studies on Ultraviolet Sensitivity in Silkworm, with Special Reference to Variations in Its Killing Effect during the Larval Instar Stage. Jpn. J. Appl. Entomol. Zool..

[30] Lah E.F.C., Musa R.N.A.R., Ming H.T. (2012). Effect of germicidal UV-C light (254 nm) on eggs and adult of house dustmites, *Dermatophagoides pteronyssinus* and *Dermatophagoides farinae* (Astigmata: Pyroglyhidae). Asian Pac. J. Trop. Biomed..

[31] Hori M., Shibuya K., Sato M., Saito Y. (2014). Lethal effects of short-wavelength visible light on insects. Sci. Rep..

[32] Yu H., Zhou B., Meng J., Xu J., Liu T.X., Wang D. (2017). Recombinant *Helicoverpa armigera* nucleopolyhedrovirus with arthropod-specific neurotoxin gene RjAa17f from *Rhopalurus junceus* enhances the virulence against the host larvae. Insect Sci..

[33] Yu H., Meng J., Xu J., Liu T.-X., Wang D. (2015). A Novel Neurotoxin Gene ar1b Recombination Enhances the Efficiency of *Helicoverpa armigera* Nucleopolyhedrovirus as a Pesticide by Inhibiting the Host Larvae Ability to Feed and Grow. PLoS ONE.

[34] El-Salamouny S., Ranwala D., Shapiro M., Shepard B.M., Farrar R.R. (2009). Tea, Coffee, and Cocoa as ultraviolet radiation protectants for the beet armyworm nucleopolyhedrovirus. J. Econ. Entomol..

[35] Shapiro M., El Salamouny S., Merle Shepard B. (2008). Green tea extracts as ultraviolet protectants for the beet armyworm, *Spodoptera exigua* nucleopolyhedrovirus. Biocontrol Sci. Technol..

[36] McIntosh A.H., Grasela J.J., Lua L., Braunagel S.C. (2004). Demonstration of the protective effects of fluorescent proteins in baculoviruses exposed to ultraviolet light inactivation. J. Insect Sci..

[37] Grasela J.J., McIntosh A.H., Ignoffo C.M., Goodman C.L. (2002). Insect cells and their potential as stabilization barriers for DNA of multiple and single nucleopolyhedroviruses against ultraviolet-B simulated sunlight inactivation. In Vitro Cell. Dev. Biol. Anim..

[38] Van Houte S., van Oers M.M., Han Y., Vlak J.M., Ros V.I.D. (2015). Baculovirus infection triggers a positive phototactic response in caterpillars: A response to Dobson et al., (2015). Biol. Lett..

[39] Benesh D.P., Duclos L.M., Nickol B.B. (2005). The behavioral response of amphipods harboring *Corynosoma constrictum* (Acanthocephala) to various components of light. J. Parasitol..

[40] Johansen N.S., Vänninen I., Pinto D.M., Nissinen A.I., Shipp L. (2011). In the light of new greenhouse technologies: Direct effects of artificial lighting on arthropods and integrated pest management in greenhouse crops. Ann. Appl. Biol..

[41] Sun Y.X., Tian A., Zhang X.B., Zhao Z.G., Zhang Z.W., Ma R.Y. (2014). Phototaxis of *Grapholitha molesta* (Lepidoptera: Olethreutidae) to different light sources. J. Econ. Entomol..

[42] Singh A.K., Saxena K.N. (2004). Attraction of larvae of the armyworm *Spodoptera litura* (Lepidoptera: Noctuidae) to coloured surfaces. Eur. J. Entomol..

[43] Tokushima Y., Uehara T., Yamaguchi T., Arikawa K., Kainoh Y., Shimoda M. (2016). Broadband photoreceptor are involved in violet light preference in the parasitoid fly *Exorista japonica*. PLoS ONE.

[44] Castrejon F., Rojas J.C. (2011). Behavioral responses of larvae and adults of *Estigmene acrea* (Lepidoptera: Arctiidae) to light of different wavelengths. Fla. Entomol..

[45] Otsuna H., Shinomiya K., Ito K. (2014). Parallel neural pathways in higher visual centers of the *Drosophila* brain that mediate wavelength-specific behavior. Front. Neural Circuits.

[46] Gorostiza E.A., Colomb J., Brembs B. (2016). A decision underlies phototaxis in an insect. Open Biol..

[47] Yamaguchi S., Desplan C., Heisenberg M. (2010). Contribution of photoreceptor subtypes to spectral wavelength preference in *Drosophila*. Proc. Natl. Acad. Sci. USA.

[48] Daiker V., Häder D.-P., Richter P.R., Lebert M. (2011). The involvement of a protein kinase in phototaxis and gravitaxis of *Euglena gracilis*. Planta.

[49] Häder D.-P., Iseki M., Schwartzbach S.D., Shigeoka S. (2017). Photomovement in Euglena. Euglena: Biochemistry, Cell and Molecular Biology.

[50] Sugimoto Y., Nakamura H., Ren S., Hori K., Masuda S. (2017). Genetics of the Blue Light-Dependent Signal Cascade That Controls Phototaxis in the *Cyanobacterium Synechocystis* sp. PCC6803. Plant Cell Physiol..

[51] Muntz W.R.A. (1963). The Development of Phototaxis in the Frog (*Rana Temporaria*). J. Exp. Biol..

[52] Kitabatake S., Shimizu I., Kato M. (1983). Wavelength-dependent properties of phototaxis in larvae of *Bombyx mori*. Photochem. Photobiol..

