# Supplementary material for: Effect of Different Light Spectrum in Helicoverpa armigera Larvae during HearNPV Induced Tree-Top Disease
Source: Insects. 2018 Dec 4;9(4):183. doi: 10.3390/insects9040183 (PMC6316081; doi:10.3390/insects9040183)
Supplement: Supplementary file 1 [file insects-09-00183-s001.pdf]

insects

Supplementary Materials

Article

Effect of Different Light Spectrum in *Helicoverpa armigera* Larvae during HearNPV Induced Tree-Top Disease

Mandira Katuwal Bhattarai [1,†], Upendra Raj Bhattarai [1,†], Ji-nian Feng [2,*] and Dun Wang [1,*]

[1] State Key Laboratory of Crop Stress Biology for Arid Areas, Northwest A&F University, Yangling 712100, China; mandirakat123@gmail.com (M.K.B.); upendrarajbhattarai@gmail.com (U.R.B.)
[2] Department of Entomology, Northwest A&F University, Yangling 712100, China
* Correspondence: jinianf@nwsuaf.edu.cn (J.F.); wanghande@nwsuaf.edu.cn (D.W.); Tel.: +86-29-8709-1511 (D.W.)
† These authors contributed equally.

Received: 9 October 2018; Accepted: 20 November 2018; Published: date

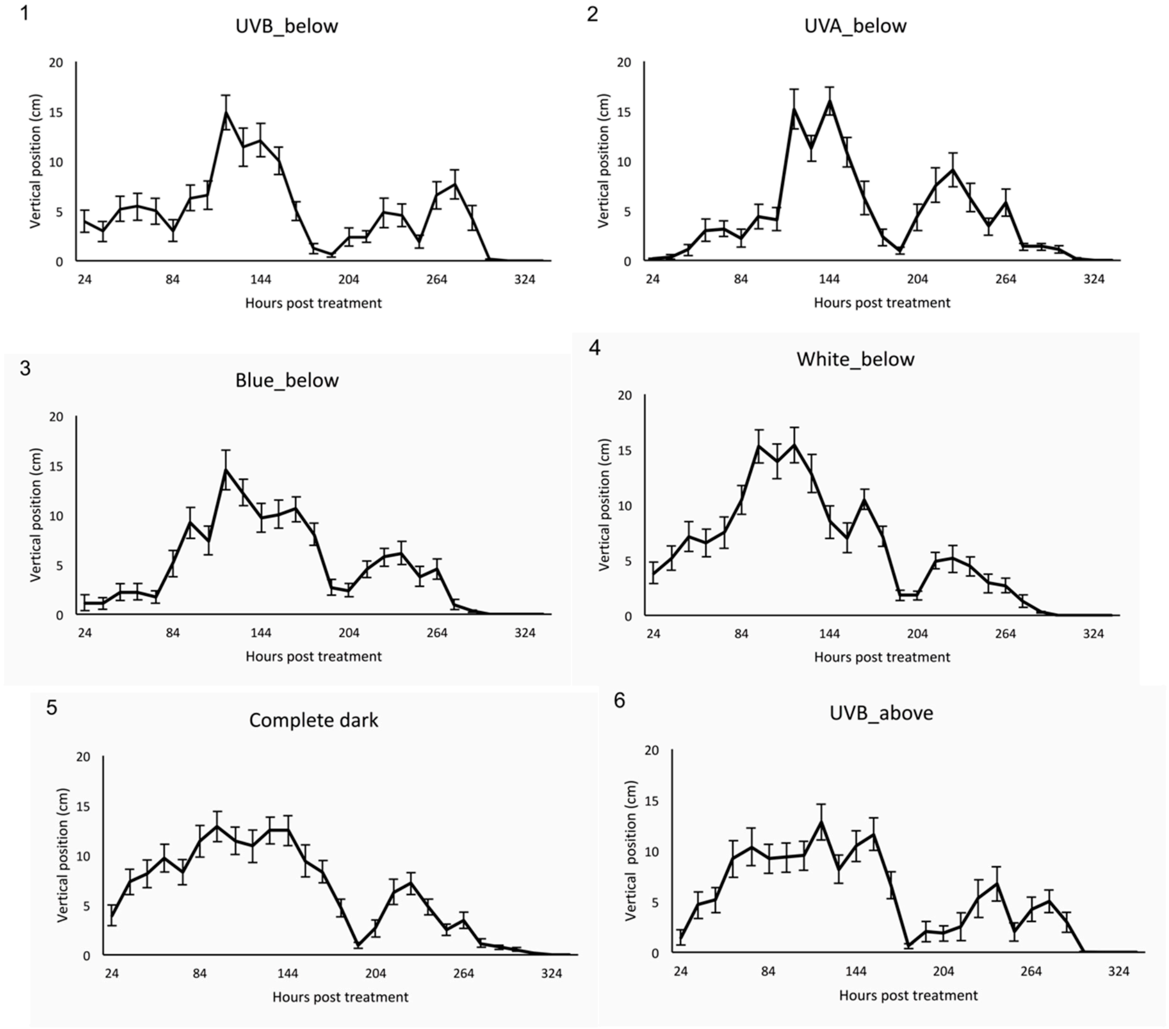

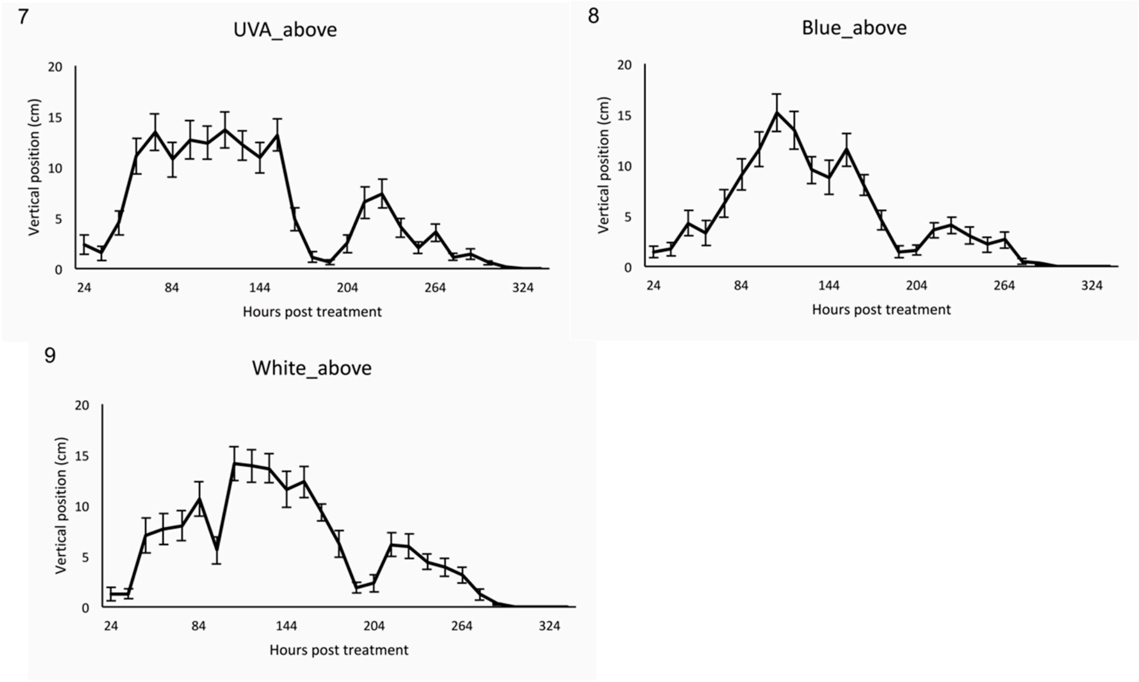

Figure S1–S9. Climbing behavior of uninfected larvae in different light treatments. Y-axis represents the vertical position of the larvae in centimeter from the base of the cylinder; X-axis represents different hour post light treatments until pupation; Error bar represents standard error mean (SEM); S1–S4 illustrates larval climbing behavior in different light treatments from below (L: D, 12h: 12h); S5 during complete dark (L: D, 0h: 24h); and S6–S9 during different light treatments from above (L: D, 12h: 12h) as mentioned in the title of each line graph. .

© 2018 by the authors. Licensee MDPI, Basel, Switzerland. This article is an open access article distributed under the terms and conditions of the Creative Commons Attribution (CC BY) license (http://creativecommons.org/licenses/by/4.0/).
